# Association between serum vitamin D status and the anti-seizure treatment in Chinese children with epilepsy

**DOI:** 10.3389/fnut.2022.968868

**Published:** 2022-08-29

**Authors:** Na Dong, Hong-Li Guo, Ya-Hui Hu, Jiao Yang, Min Xu, Le Ding, Jin-Chun Qiu, Zhen-Zhou Jiang, Feng Chen, Xiao-Peng Lu, Xiao-Nan Li

**Affiliations:** ^1^Pharmaceutical Sciences Research Center, Department of Pharmacy, Children’s Hospital of Nanjing Medical University, Nanjing, China; ^2^Institute of Pharmaceutical Science, China Pharmaceutical University, Nanjing, China; ^3^Department of Information Science, Children’s Hospital of Nanjing Medical University, Nanjing, China; ^4^Department of Neurology, Children’s Hospital of Nanjing Medical University, Nanjing, China; ^5^Department of Children Health Care, Children’s Hospital of Nanjing Medical University, Nanjing, China

**Keywords:** epilepsy, vitamin D, anti-seizure medications, children, nutrition

## Abstract

**Objective:**

To compare the serum 25-OH-VitD levels, the major marker of vitamin D (VitD) status, between healthy children and children with epilepsy before initiation of and during anti-seizure medications (ASMs) treatment and to evaluate the potential influence factors on 25-OH-VitD levels. Another major aim was to assess the potential role of VitD supplementation.

**Methods:**

For comparison, we finally enrolled and collected data from 6,338 healthy children presenting to Health Care Department and 648 children visiting primary care pediatricians with symptoms of epilepsy in Children’s Hospital of Nanjing Medical University from January 2019 to June 2021. The demographic and biochemical characteristics of each child were extracted from the hospital information system.

**Results:**

Serum 25-OH-VitD levels in 648 children with epilepsy were significantly lower than those of 6,338 healthy children (*P* < 0.0001), and the percentage of VitD insufficiency and deficiency status in pediatric patients was 49.19%. Of note, the serum 25-OH-VitD levels in children with newly diagnosed epilepsy before receiving any ASMs treatment were also significantly lower than those in healthy controls. Interestingly, ASMs therapy, alone or in combination, did not consistently reduce baseline serum VitD levels in children with epilepsy. The lower serum VitD levels in pediatric patients than those in healthy children might be related to the disease itself, rather than the ASMs treatment. As expected, VitD supplementation substantially increased the serum 25-OH-VitD levels (*P* < 0.0001). More critically, children with epilepsy receiving VitD supplementation achieved good seizure control in our study.

**Significance:**

In this retrospective study, the childhood epilepsy before initiation of and during ASMs treatment decreased the serum 25-OH-VitD concentrations, suggesting a clear association between epileptic disease and the risk of VitD deficiency. ASMs coadministration and long-term valproic acid treatment did not worse VitD-deficiency status, but in the small group receiving VitD supplementation, there was a significant improvement in reduction of seizure frequency. Therefore, pediatric clinicians are urged to raise public awareness of epilepsy-associated VitD deficiency.

## Highlights

- Deficiency or insufficiency of serum VitD levels in healthy children is uncommon.
- Serum VitD levels in children with epilepsy were significantly lower than those in healthy children, and the deficiency or insufficiency status was more common.
- ASMs therapy, alone or in combination, did not continuously worse baseline serum VitD levels in children with epilepsy. The lower serum VitD levels in pediatric patients than in healthy children might be related to the disease itself, rather than the ASMs treatment.
- Direct VitD supplementation significantly corrected the serum VitD levels, and was critically associated with a reduction in epileptic activity in those children.
- Pediatric clinicians are urged to heighten public awareness regarding epilepsy-associated VitD deficiency or insufficiency.

## Introduction

Vitamin D (VitD) is a key nutrient for both healthy children and those with chronic illnesses (1) due to its wide spectrum of skeletal and extra-skeletal activities (2). In 2020, the Endocrine Society of America defined VitD sufficiency as serum 25-OH-VitD levels (the major marker of VitD status) above 50 nmol/L (20 ng/ml), insufficiency as serum 25-OH-VitD levels between 30 and 50 nmol/L, and deficiency as serum 25-OH-VitD concentration below 30 nmol/L (12 ng/ml), respectively (3). There is consensus that severe VitD deficiency should be corrected (2, 4). In fact, hypovitaminosis D is a global health problem (5). And the complexities of VitD metabolism (6, 7) pose difficulties in the identification and determination of factors related to VitD deficiency. Actually, its status is largely determined by environmental and behavioral factors such as diet, physical activity, physical fitness, geographical location, seasonality, socioeconomic status, and by others like age, sex, genetics, body composition (8, 9).

More than four decades ago, Offermann et al. (10) first reported the high prevalence of low VitD status among children with epilepsy. Recently, hypovitaminosis D was also reported in children with epilepsy receiving anti-seizure medications (ASMs), especially enzyme-inducing ASMs (EIASMs) (11–13). EIASMs exert their effects on destructing VitD status via inducing cytochrome P450 (14). However, it still remains controversial over the effects of valproic acid (VPA) – a type of non-enzyme-inducing ASMs (NEIASMs), on the VitD levels. Some studies suggested that VPA monotherapy had a negative effect (15, 16), but others denied that VitD levels could be affected by VPA treatment (17). Additionally, treatment duration, mono- or add-on therapy, and types of ASMs could also affect VitD status (18–20). Even brain lesions also have an influence on VitD status (17). However, the sample sizes in healthy children and children with epilepsy before initiation of and during ASMs treatment were small in previous studies, and few studies compared the VitD levels in those groups of study participants at the same time (21). Interestingly, patients with epilepsy after VitD supplementation were associated with well-controlled seizures in a pilot study (22). However, some reports (20) hold the opposite point that VitD made no difference in improving epilepsy seizure.

To bring clarity to this issue, this study aimed to compare the serum 25-OH-VitD status in healthy children and children with epilepsy before initiation of and after ASMs treatment and to investigate the potential risk factors on VitD deficiency and insufficiency levels. And another aims of our study were to examine the longitudinal change of VitD levels and to assess the response to ASMs during VitD supplementation.

## Materials and methods

### Study population

**FIGURE 1:**
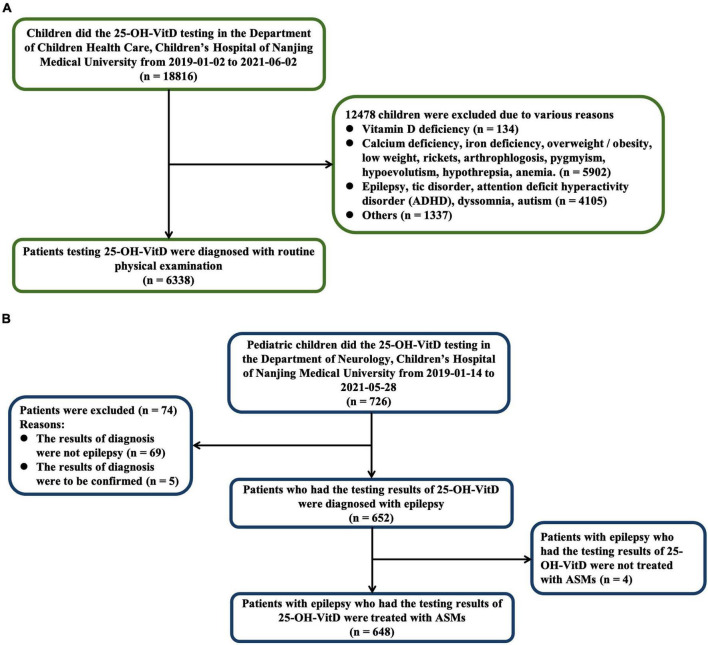
Numbers of healthy children **(A)** and children with epilepsy **(B)** who were eligible for the study.

This case-cohort study was conducted on individuals who developed childhood epilepsy and a cohort of healthy subjects between the ages of 0 and 18 years old. The control group were recruited from January 2, 2019 to June 2, 2021, who did regular physical examination including items at least such as serum 25-OH-VitD levels in the Department of Child Health Care, Children’s Hospital of Nanjing Medical University (**The geographical coordinates of Nanjing are 118°22′ – 119°14′E, 31°14′ – 32°37′N**). We excluded those children with various diagnosis including VitD deficiency, calcium deficiency, overweight/obesity, low weight, rickets, arthrophlogosis, pygmyism, hypoevolutism, hypothrepsia, anemia, epilepsy, tic disorder, attention deficit hyperactivity disorder, dyssomnia, autism, and so on. A case was defined as a child who received ASMs, with an epilepsy diagnosis and had serum 25-OH-VitD levels measured from January 14, 2019 to May 28, 2021 in the Department of Neurology of the same hospital. The inclusion and exclusion criteria are listed in Figure 1.

### Definitions

Baseline 25-OH-VitD values were obtained from those children who had just been diagnosed with epilepsy, but had not started any ASMs treatment, and just had serum vitamin levels measured during January 14, 2019 to June 1, 2021 in the Department of Neurology of our hospital. Children with epilepsy showing poor tolerance to two or more ASMs and failing to achieve sustained seizure freedom with good medication adherence, was regarded as the drug-resistant epilepsy (23). According to the guideline of the Endocrine Society of America, (3) serum VitD status were considered to be replete (serum 25-OH-VitD ≥ 50 nmol/L), insufficient (serum 25-OH-VitD ≥ 30 – < 50 nmol/L), and deficient (serum 25-OH-VitD < 30 nmol/L), respectively. Additionally, we defined stages of childhood as infancy (ages of 28–364 days), early childhood (ages of 1–6 years), middle childhood (ages of 6–12 years), and adolescence (ages of 12–18 years), respectively (24). The ASMs were categorized and analyzed according to type of ASMs (EIASMs or NEIASMs, Table 1). Patients who used one or more EIASMs were included in the EIASMs group, regardless of whether they used NEIASMs or not. Seizure-free was defined as absence of seizures on unchanged medications, while seizure was defined as patients with < 50% reduction in seizure frequency on unchanged medications.

**TABLE 1:** ASMs used by the participants with epilepsy.

Enzyme-inducing ASMs	Non-enzyme-inducing ASMs
Oxcarbazepine	Clonazepam
Perampanel	Lacosamide
Phenytoin	Lamotrigine
Topiramate (≥ 200 mg/day)	Levetiracetam
	Topiramate (<200 mg/day)
	Valproate
	Vigabatrin

### Anti-seizure treatment protocols

Pediatric patients included in this study were all treated according to the National Medical Products Administration-approved indications. All real-world clinical treatment protocols were retrospectively collected to evaluate the potential roles in the serum 25-OH-VitD status. Collectively, seven ASMs treatment protocols (i.e., AST-P1 to AST-P7) were summarized as below, including VPA monotherapy and VPA combination with other ASMs.

(1) AST-P1: VPA monotherapy

(2) AST-P2: VPA + EIASMs + Other NEIASMs

(3) AST-P3: VPA + EIASMs

(4) AST-P4: VPA + Other NEIASMs

(5) AST-P5: EIASMs + NEIASMs excluding VPA

(6) AST-P6: EIASMs

(7) AST-P7: NEIASMs excluding VPA

### Clinical data collection

Clinical data were obtained from the hospital information system (HIS) using a standard *pro forma* sheet, which included various data as below:

1. Demographic data of potential influencing factors on VitD status: age, sex, body weight (BW), and physical examination time.
2. Epilepsy history: patient-reported outcomes, seizure outcome, seizure manifestation, ASMs regimen (EIASMs) including oxcarbazepine, phenytoin, perampanel, and topiramate (≥ 200 mg/day); (25) and NEIASMs including VPA, lamotrigine, levetiracetam, lacosamide, clonazepam, vigabatrin and topiramate (< 200 mg/day), (25) number of ASMs the patient was on, duration and dosage on VPA treatment, and the concentration monitoring data of VPA.
3. Brain magnetic resonance imaging (MRI) images and findings.
4. Dosage of VitD supplementation.
5. Other demographic and clinical outcomes data were also collected from each individual patient’s medical record.

### Statistical analysis

The statistical analysis of data was performed using GraphPad Prism 9.0 (GraphPad Software, La Jolla, CA, United States) and SPSS version 26.0 software (IBM, Armonk, NY, United States). Shapiro–Wilk tests were used to assess normality. Descriptive data of subjects were presented as number (n) and percentage (%) for categorical variables, but median and interquartile (I.Q.) range for continuous variables. The Kolmogorov–Smirnov test was used to identify the differences among groups. The non-parametric Mann-Whitney test was applied to compare quantitative data in two groups. The chi-square test was applied to compare nominal data in two groups. Correlation analysis of potential affecting factors on 25-OH-VitD levels was performed by Spearman correlation. A value of *P* < 0.05 was considered statistically significant.

### Legal and ethical considerations

The study was conducted in accordance with the Helsinki Declaration. Medical data collection was approved by the Ethics Committee of the Children’s Hospital of Nanjing Medical University (Protocol number 202205055-1). Parents/guardians did not provide informed consent owing to the retrospective nature of this study.

## Results

### Characteristics and status of included pediatric subjects

A total of 6,986 pediatric subjects were retained in our final study cohort according to the inclusion and exclusion criteria. These included 6,338 healthy children (Figure 1A) and 648 children with epilepsy, respectively (Figure 1B). The characteristics of both the healthy group and epileptic group are presented in Table 2. The groups differed in size, but were similar according to age, sex, and body weight.

**TABLE 2:** Demographic characteristics of healthy children and pediatric patients with epilepsy.

	Healthy children	Children with epilepsy
*N*	6,338	648
Records of testing 25-OH-VitD	6,338	1,112
Age (years)	5 (3.08 – 8)	6.17 (3.42 – 8.83)
Growth stage (n, %)		
Infancy (29 – 364 days)	6 (0.09)	51 (4.59)
Early childhood (1 – 6 years)	4,171 (65.81)	495 (44.51)
Middle childhood (6 – 12 years)	1,852 (29.22)	436 (39.21)
Adolescence (12 – 18 years)	309 (4.88)	130 (11.69)
Sex (n, %)		
Male	3,731 (58.87)	356 (54.94)
Female	2,607 (41.13)	292 (45.06)
Weight (kg)	17 (12 – 24)	25 (17 – 36)
25-OH-VitD (nmol/L, n, %)	72.55 (56.32 – 88.69)	50.47 (38.64 – 68.13)
<30 nmol/L	99 (1.56)	134 (12.05)
≥30 – < 50 nmol/L	979 (15.45)	413 (37.14)
≥50 nmol/L	5,260 (82.99)	565 (50.81)
Response to ASMs (n, %)		
Seizure free		776 (69.78)
Seizure		275 (24.73)
Partial seizure		61 (5.49)
Anti-seizure treatment (n, %)		
Number of ASMs		
1 ASM		579 (52.07)
2 ASMs		368 (33.09)
≥3 ASMs		161 (14.48)
Type of ASMs		
CZP		90
LCM		61
LEV		418
LTG		115
OXC		286
PER		8
PHT		1
TPM		111
VGB		6
VPA		741
MRI		302
Abnormal		168 (55.63)
Normal		134 (44.37)
Vitamin D (VitD) supplement (n, %)		
With VitD supplement		100 (9.00)
Without VitD supplement		1,012 (91.00)
VitD intake (IU/day)		700 (100 – 800)

### Vitamin D status in healthy children and pediatric patients with epilepsy

**FIGURE 2:**
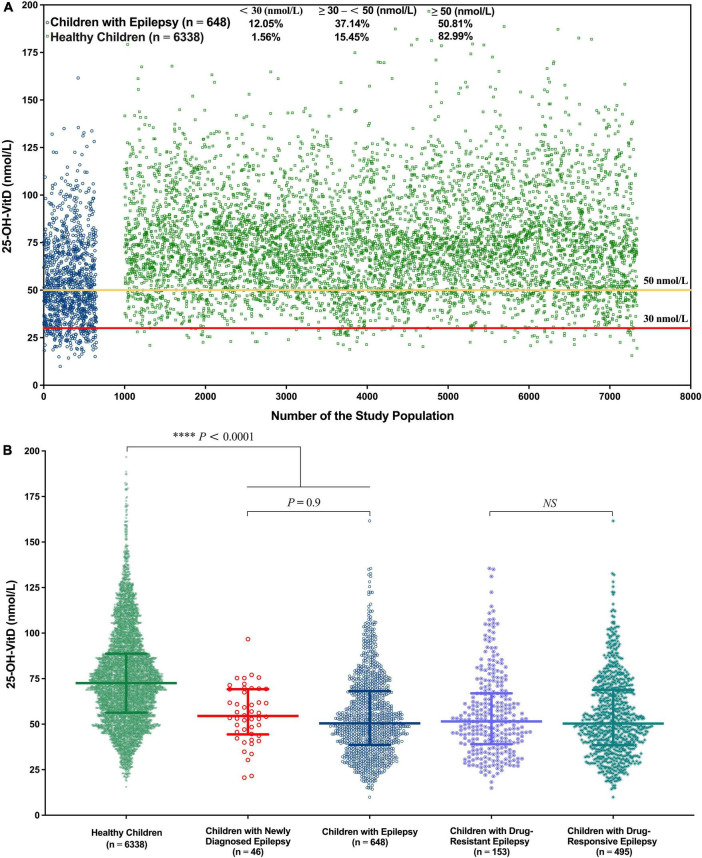
Overall serum 25-OH-VitD status of 6,338 health children and 648 children with epilepsy. **(A)** Serum 25-OH-VitD status of 6,338 health children and 648 children with epilepsy and proportion of VitD sufficiency, insufficiency and deficiency. **(B)** Comparison of healthy children, children with epilepsy, children with newly diagnosed epilepsy (before ASMs treatment), children with drug-resistant epilepsy and children with drug-responsive epilepsy in serum 25-OH-VitD status.

The median serum 25-OH-VitD levels in healthy children and children with epilepsy were 72.55 (I.Q. range, 56.32–88.69) and 50.47 (I.Q. range, 38.64–68.13) nmol/L (*P* < 0.0001), respectively. In 648 pediatric patients, VitD deficiency was present in 134 lab tests (12.05%), and a further 413 lab tests (37.14%) reported VitD insufficiency, that is to say, totally, about 50% of the measured results were less than 50 nmol/L. But the percentage number of healthy controls was 1.56 and 15.45%, respectively (Figure 2A). Notably, the median serum 25-OH-VitD levels in those children with newly diagnosed epilepsy were 51.51 (I.Q. range, 40.74–67.11) nmol/L, defined as baseline values (*n* = 46), which were significantly lower than those in healthy children (*P* < 0.0001) (Figure 2B). But there was no any difference between before initiation of and during ASMs treatment for those patients (*P* = 0.9) (Figure 2B).

### Impacting factors on vitamin D status

#### Age

**FIGURE 3:**
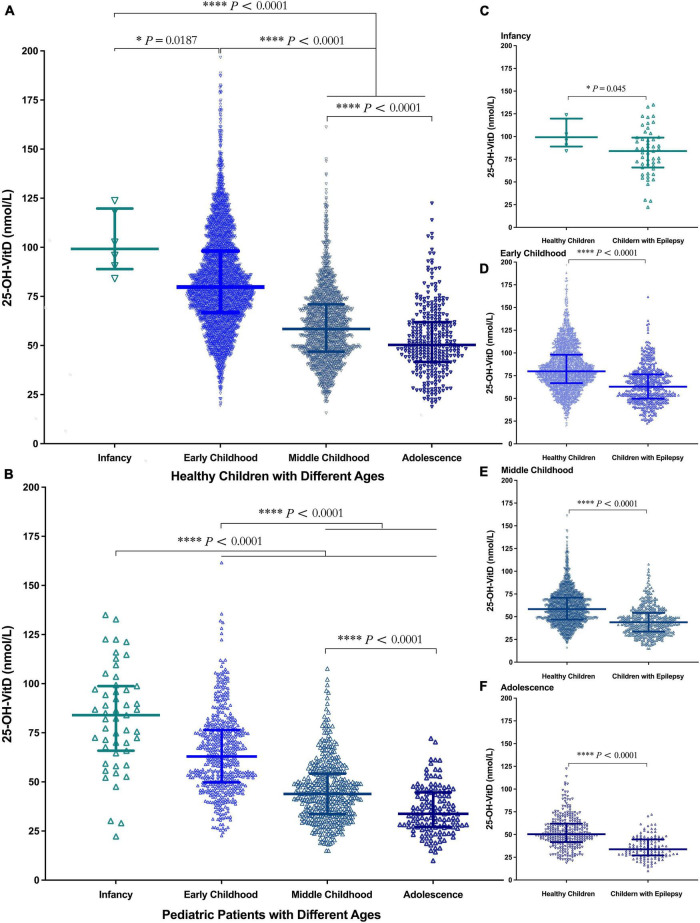
Comparison in serum 25-OH-VitD status of subjects with different ages (Infancy, Early Childhood, Middle Childhood, and Adolescence). **(A)** Comparison in serum 25-OH-VitD status among four groups of healthy children. **(B)** Comparison in serum 25-OH-VitD status among four groups of children with epilepsy. **(C)** Comparison in serum 25-OH-VitD status between healthy children and children with epilepsy during the infancy. **(D)** Comparison in serum 25-OH-VitD status between healthy children and children with epilepsy during the early childhood. **(E)** Comparison in serum 25-OH-VitD status between healthy children and children with epilepsy during the middle childhood. **(F)** Comparison in serum 25-OH-VitD status between healthy children and children with epilepsy during the adolescence.

Possible age-related effects were assessed by separating the subjects into four age groups: infancy, early childhood, middle childhood, and adolescence. Regardless of children with epilepsy or their controls, the serum 25-OH-VitD levels showed a continuous downward trend from infancy to adolescence. Of note, the median serum 25-OH-VitD levels declined approximately 1 and 1.5 folds from infancy to adolescence in healthy and epileptic children, respectively (Figures 3A,B). It needs to be emphasized again that, in all four age groups, the serum 25-OH-VitD levels in healthy children were significantly higher than those in children with epilepsy (Figures 3C–F). In addition, a Spearman analysis highlighted the significant negative correlation between age and serum 25-OH-VitD levels among healthy subjects (*r* = −0.6, *P* < 0.0001). A similar finding was observed in children with epilepsy (*r* = −0.7, *P* < 0.0001, Supplementary Figure 1).

#### Sex

**FIGURE 4:**
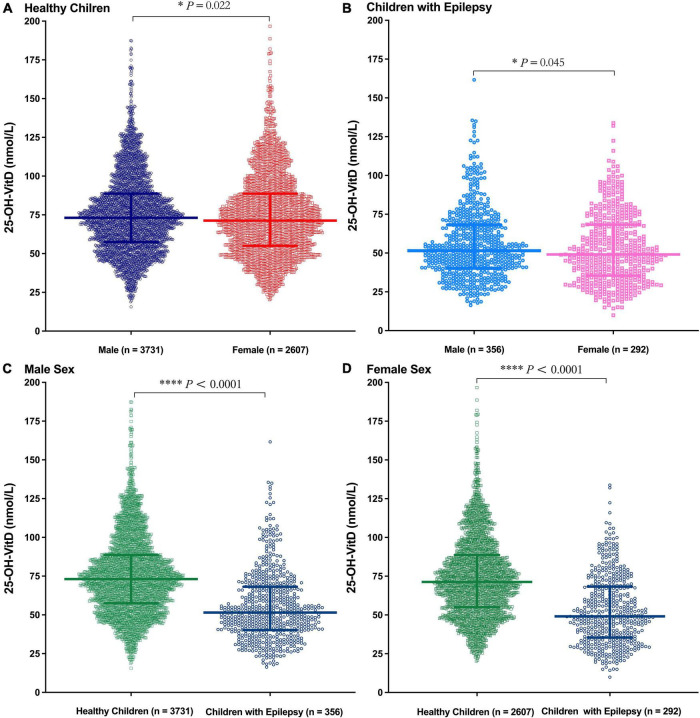
Comparison in serum 25-OH-VitD status of subjects with different sex (Male and Female). **(A)** Comparison in serum 25-OH-VitD status between males and females of healthy children. **(B)** Comparison in serum 25-OH-VitD status between males and females of children with epilepsy. **(C)** Comparison in serum 25-OH-VitD status between healthy children and children with epilepsy among male sex. **(D)** Comparison in serum 25-OH-VitD status between healthy children and children with epilepsy among female sex.

Significant sex differences in serum 25-OH-VitD levels occurred in both healthy children (Figure 4A, *P* = 0.022) and epileptic children (Figure 4B, *P* = 0.045). Moreover, the serum 25-OH-VitD levels of male healthy controls were significantly higher than those of male children with epilepsy (Figure 4C, *P* < 0.0001). The same was true in females (Figure 4D, *P* < 0.0001). Besides, males had higher serum 25-OH-VitD concentrations than females (*P* < 0.0001) in middle childhood for the healthy controls (Supplementary Figure 2C). Moreover, this phenomenon was also seen in middle childhood and adolescence of cases (Supplementary Figures 2G,H). However, there were no significant changes in another age period (i.e., infancy and early childhood) of both the control and case groups (Supplementary Figure 2).

#### Body weight

**FIGURE 5:**
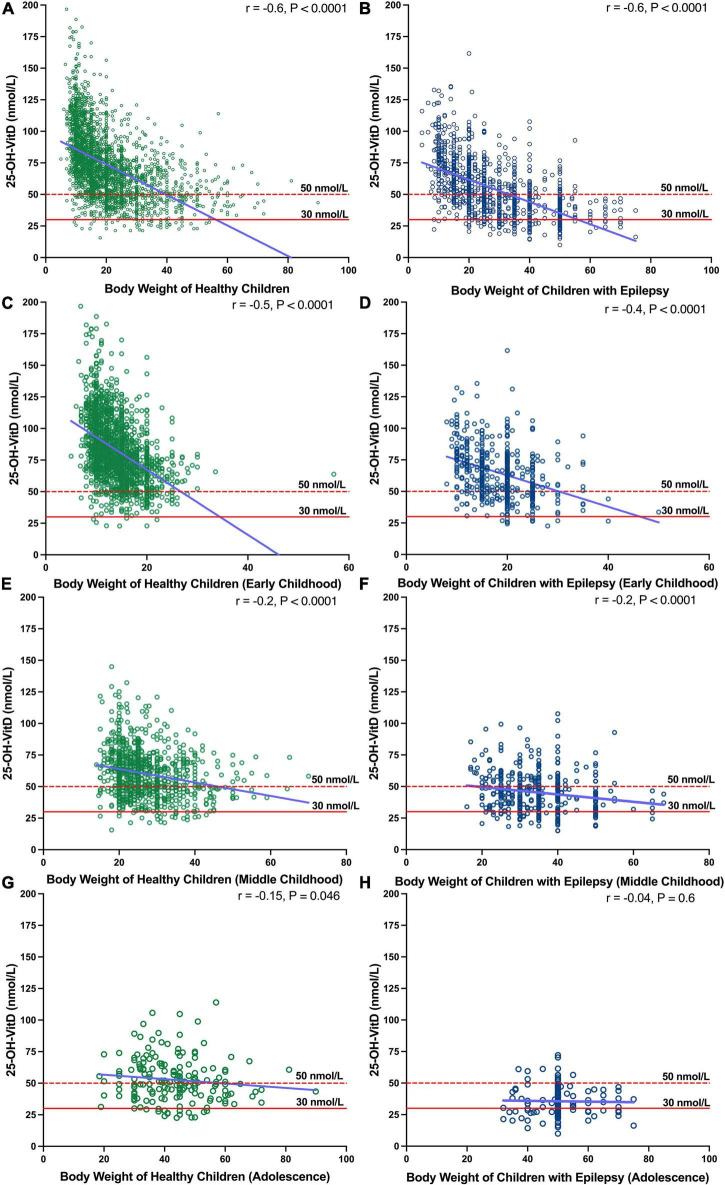
Association between BW and serum 25-OH-VitD status in healthy subjects and epileptic subjects with different ages (Early Childhood, Middle Childhood, and Adolescence). **(A)** Association between BW and serum 25-OH-VitD status in healthy subjects. **(B)** Association between BW and serum 25-OH-VitD status in epileptic subjects. **(C)** Association Early childhood in healthy children. **(D)** Early childhood in children with epilepsy. **(E)** Middle childhood in healthy children. **(F)** Middle childhood in children with epilepsy. **(G)** Adolescence in healthy children. **(H)** Adolescence in children with epilepsy.

The Spearman analysis showed that there was negative association between BW and the serum 25-OH-VitD levels in both healthy children and children with epilepsy (*r* = −0.6, *P* < 0.0001, Figures 5A,B). This significant association was more prominent in early childhood (*r* = −0.5, *P* < 0.0001; *r* = −0.4, *P* < 0.0001, Figures 5C,D) but not in other age groups (Figures 5E–H).

#### Season

**FIGURE 6:**
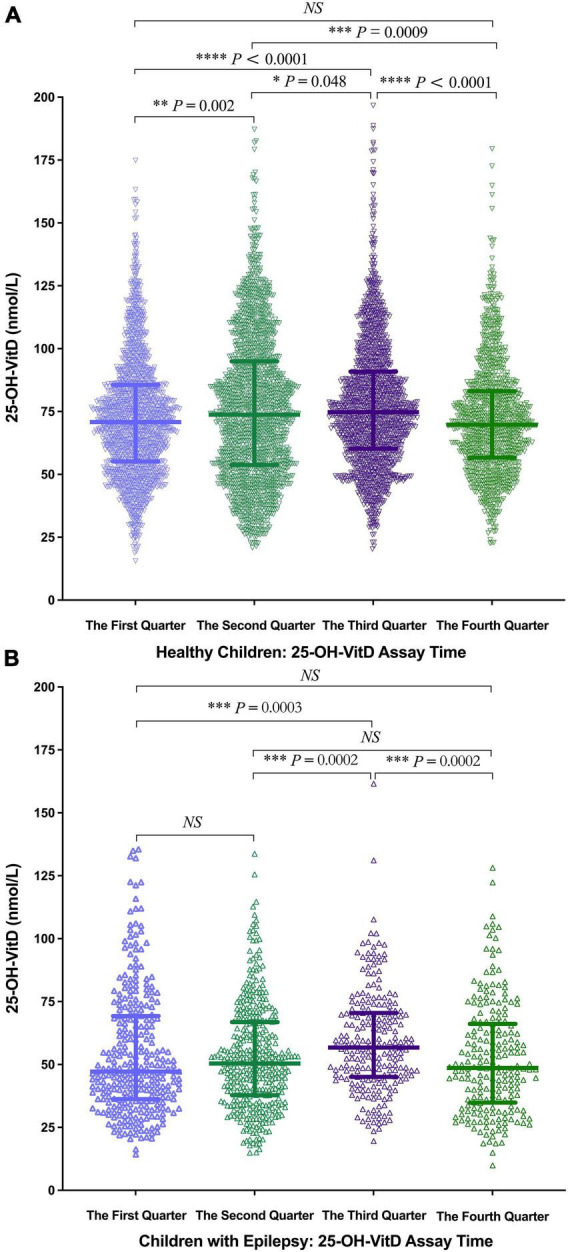
Comparison in serum 25-OH-VitD status of subjects with different assay time. **(A)** Comparison in serum 25-OH-VitD status with different assay time of healthy children. **(B)** Comparison in serum 25-OH-VitD status with different assay time of children with epilepsy.

Seasonal differences in serum 25-OH-VitD levels were found in both healthy children (Figure 6A) and children with epilepsy (Figure 6B). The highest was in the 3rd quarter (median 74.78 vs. 56.99 nmol/L), followed by the 2nd (median 73.74 vs. 50.55 nmol/L) and 4th quarter (median 69.77 vs. 48.68 nmol/L), and the lowest was in the 1st quarter (median 70.81 vs. 47.23 nmol/L). Of note, serum 25-OH-VitD levels differed most between the two groups (i.e., 1.5 folds) in the first quarter.

#### Anti-seizure treatment

##### Treatment protocols

**FIGURE 7:**
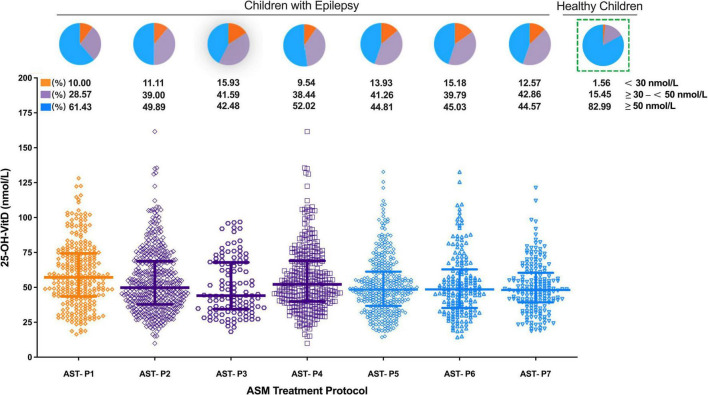
Comparison in serum 25-OH-VitD status of children with epilepsy with different ASM treatment protocols and proportion of VitD sufficiency, insufficiency and deficiency (including healthy children).

As shown in Figure 7, serum 25-OH-VitD levels were also associated with the ASMs treatments. Children with epilepsy receiving VPA monotherapy showed significantly higher serum 25-OH-VitD concentrations than those taking VPA with other ASMs (APT-P1 vs. APT-P2, 56.94 vs. 49.80 nmol/L, *P* = 0.001). In particular, serum 25-OH-VitD levels were significantly decreased (median 48.39 vs. 56.94 nmol/L, *P* < 0.0001) in children receiving AST-P5 treatment, a therapy without VPA, than those who were administered VPA alone (APT-P1). Moreover, there was a clear downward trend in serum 25-OH-VitD levels (*P* = 0.049) when we compared AST-P2 and AST-P5 regimens. The major difference between the two protocols was that the former included VPA, while the latter did not.

Looking from another aspect, a significant change in serum 25-OH-VitD levels was noted between the EIASMs and NIASMs (AST-P3 vs. AST-P4; median 44.03 vs. 52.14 nmol/L, *P* = 0.02), but there was no significance between the EIASMs and NIASMs excluding VPA (AST-P6 vs. AST-P7; median 48.54 vs. 48.11 nmol/L). Overall, children receiving VPA alone had the highest serum 25-OH-VitD levels, by comparison with any other ASMs treatment protocols, where the VitD levels reduced to varying degrees (P_AST–P1 vs. AST–P3_ = 0.0002, P_AST–P1 vs. AST–P4_ = 0.015, P_AST–P1 vs. AST–P6_ < 0.0001, P_AST–P1 vs. AST–P7_ < 0.0001). In addition, no significant difference in serum 25-OH-VitD concentrations was found between AST-P3 and AST-P4. But in the NEIASMs group, with or without VPA, there was a significant difference in serum 25-OH-VitD levels between AST-P4 and AST-P7 (*P* = 0.016).

##### VPA treatment duration

**FIGURE 8:**
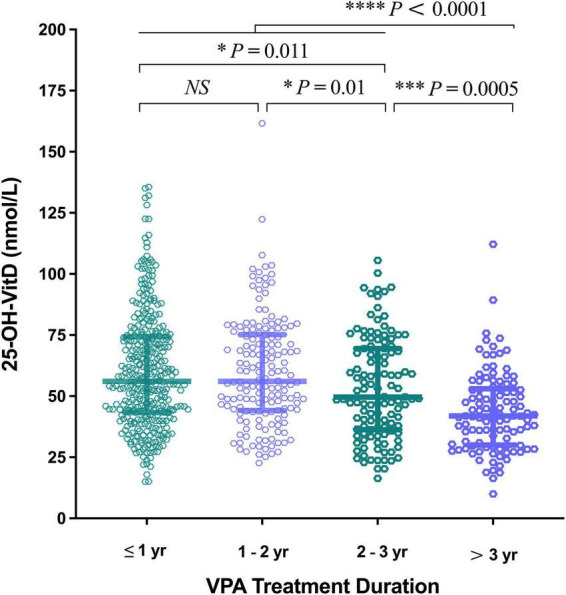
Comparison in serum 25-OH-VitD status of children with epilepsy on valproic acid (VPA) therapy duration.

A more significantly negative impact on the serum 25-OH-VitD levels occurred in the longer duration of VPA treatments (> 3 years, median = 41.86 nmol/L; > 2 – ≤ 3 years, median = 49.61 nmol/L), compared to shorter ones (≤ 1 year, median 56.00 nmol/L; > 1 – ≤ 2 years, 56.06 nmol/L) (Figure 8). Intriguingly, decline in the levels of serum 25-OH-VitD was more prominent in patients with > 3 years of therapy than those with > 2 – ≤ 3 years of therapy duration (*P* = 0.0005). However, there was no any difference among patients with shorter duration of VPA treatment (≤ 1 year and 1–2 years).

#### Brain magnetic resonance imaging

**FIGURE 9:**
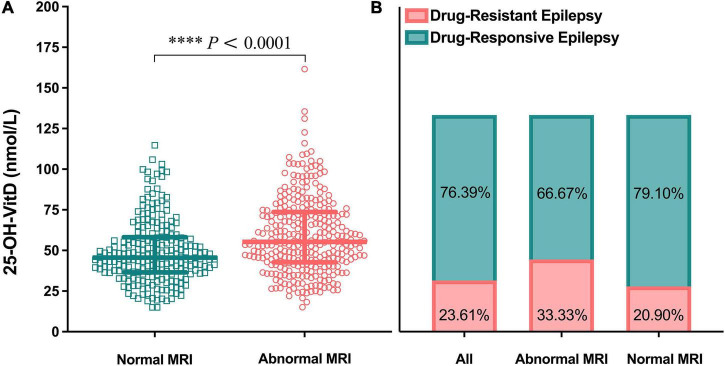
Comparison in serum 25-OH-VitD status of children with epilepsy of different brain magnetic resonance imaging (MRI) findings. **(A)** Comparison in serum 25-OH-VitD status between abnormal and normal brain MRI findings group. **(B)** Proportion of children with drug-resistant epilepsy and drug-responsive epilepsy of all pediatric patients with epilepsy, patients with abnormal brain MRI findings and patients with normal brain MRI findings.

A total of 302 pediatric patients with epilepsy had MRI records. The serum 25-OH-VitD levels in patients (*n* = 134) with normal MRI images were found to be significantly lower than those children (*n* = 168) with abnormal findings (median 45.60 vs. 55.26 nmol/L, *P* < 0.0001; Figure 9A). Furthermore, in those pediatric patients with normal MRI scans, nearly 80% experienced well-controlled epilepsy, but the remaining were drug-refractory. Similarly, the percentage number was 67 and 33% in children with abnormal MRI findings (Figure 9B), respectively. However, there were no any differences in serum 25-OH-VitD levels among children with drug-resistant and drug-responsive epilepsy (Figure 2B).

#### Vitamin D supplementation

**FIGURE 10:**
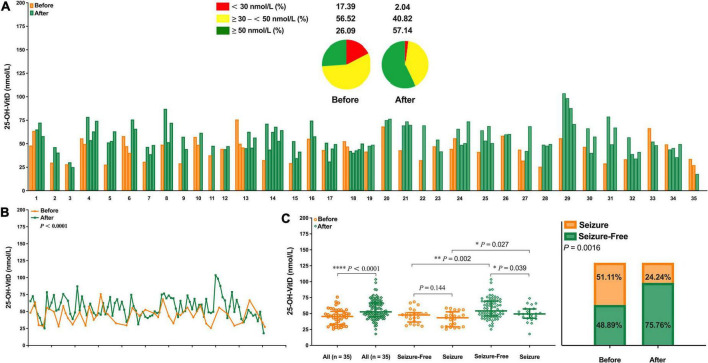
Comparison in serum 25-OH-VitD status of children with epilepsy before and after vitamin D supplementation. **(A)** 25-OH-VitD status of 35 children with epilepsy before and after vitamin D supplementation and proportion of 25-OH-VitD sufficiency, insufficiency and deficiency before and after vitamin D supplementation. **(B)** Overall comparison in serum 25-OH-VitD status of 35 children with epilepsy before and after vitamin D supplementation. **(C)** Comparison in serum 25-OH-VitD status of 35 patients before and after vitamin D supplementation and with different seizure outcomes. **(D)** Proportion of children with epilepsy seizure and seizure free before and after vitamin D supplementation.

Of the 648 children with epilepsy, only 35 were eligible as self-controls for evaluating potential roles of VitD supplementation in the seizure frequency. As shown in Figure 10A, obviously, the serum 25-OH-VitD status, insufficiency or deficiency, 74% in total, were greatly improved to be 43% after VitD supplementation. Figure 10B could more directly reflect the changes in the serum 25-OH-VitD levels before and after VitD supplementation, where the medians changed from 45.29 to 52.52 nmol/L, and increased by 16% (*P* < 0.0001) (Figure 10C). Interestingly, there was no any difference in serum 25-OH-VitD levels for patients without seizures and those still experiencing seizures before VitD supplementation (Figure 10C, *P* = 0.144). However, the difference became statistically significant after VitD supplementation (Figure 10C, *P* = 0.039). More importantly, the chi-square test showed seizure control was significantly improved after VitD supplementation, and the proportion of the seizure-free response increased from initial 49% to final 76% (*P* = 0.0016, Figure 10D).

## Discussion

This study investigated the association between the systemic VitD status of children with epilepsy and various ASMs treatments by comparing serum 25-OH-VitD levels with healthy children, and also evaluated its several determining factors like age, sex, BW, and seasonality. To be honest, correction of serum 25-OH-VitD levels by VitD supplementation was expected, but its improvement in seizures was a novel finding with great clinical value in children with epilepsy.

The first important finding of this retrospective study was that the serum 25-OH-VitD levels in children with epilepsy were much lower than those in healthy children, who had completed regular physical examination including VitD test during the same period, and that the percentage of VitD insufficiency or deficiency was also much higher (Figures 2A,B). This was in line with several previous studies (26–30). For healthy controls, geographical location and socio-economic status of Nanjing might play potential roles in the sufficient serum 25-OH-VitD levels (Figure 2A, 83%). To our knowledge, this is the largest study to date assessing VitD status in children with epilepsy (*n* = 648) on long-term ASMs therapy (18, 31, 32). Even more rarely, this is also a comparative study that simultaneously enrolled the largest number of healthy children (*n* = 6,338) (15, 29).

As well known, VitD status is largely determined by environmental, behavioral factors, and demographic factors. In this retrospective study, age, sex, BW, and seasonality became the various determinants. There was an obvious negative association between the serum 25-OH-VitD levels and age in both case and cohort groups (Supplementary Figure 1), which was in line with results from previous reports (31, 33, 34). Of note, significant decline in serum 25-OH-VitD levels was observed in adolescents among both groups of study subjects. Our findings and previous literature (32, 35–38) could corroborate each other. However, there were also studies with different results, like reports by Siddiqee et al. (39) and by McGillivray et al. (40), and VitD levels presented a considerable drop in younger age group. But the age range of their subjects, enrolled in the study, was from newborn to 5 years old. Regardless, age should be one of the most important determinants of serum VitD levels (21, 41).

The effect of sex on serum VitD is still controversial. Some reports (34, 39, 42–45) concluded that the serum 25-OH-VitD levels in males were significantly higher than those in females, but there didn’t exist any correlation for both sexes in other studies (21, 36, 46–48). In our retrospective study, notably, the effect of sex differences did occur in the two study groups observed, and it was more pronounced in the cohort group. The median 25-OH-VitD of healthy children is about 1.5 times higher than that of children with epilepsy in the same sex group (Figures 4C,D). Interestingly, these differences were more marked in school-aged and adolescent children (Supplementary Figure 2).

We observed a clear seasonal variation in serum 25-OH-VitD levels, with the highest median values observed in the 3rd quarter – a season with longest time of sunlight in the northern hemisphere (32, 49, 50). Moreover, for both study groups, serum 25-OH-VitD levels in the 3rd quarter were significantly different from any other quarter, but the results of the mutual comparisons in the other quarters were not (Figures 6A,B). Notably, the seasonal variation in serum 25-OH-VitD levels was more prominent in children of female sex than those of male sex for children with epilepsy. Such sex difference related to seasonal variation have also been reported in previous studies (51–53).

In addition, it seemed that lifestyle behaviors contribute to variable serum 25-OH-VitD levels among children with epilepsy and healthy controls. Especially, those pediatric patients are likely less physically active and less often outdoors compared to healthy controls, thereby receiving less sunlight exposure.

Another relevant finding of our study was that children with epilepsy were associated with significant reductions in serum 25-OH-VitD levels, which was in line with a previous report, (54) but ASMs treatments did not sacrifice the initial status of VitD measured before initiation of the medications (Figure 2B). Offerman et al. (10) first reported the high prevalence of lower VitD status in childhood epilepsy in 1979. Since then, such issues have received widespread attention, and similar findings have been reported (55–57). Although most studies suggest that ASMs eroded serum VitD levels, it is far from reaching consistent conclusion. In fact, VitD deficiency or insufficiency in pediatric patients is an overlooked issue among neurologists and pediatricians. In the current study, fortunately, medical records of 46 patients could be used as the baseline (i.e., median 51.51 nmol/L) for serum 25-OH-VitD concentrations. Overall, children in treatment with ASMs, their serum 25-OH-VitD levels did not change significantly from those before starting medications, regardless of the patients’ clinical response, and no any downward trend was observed (Figure 2B).

However, other studies (19, 58) concluded that the serum 25-OH-VitD levels decreased significantly compared to that at the start of ASMs therapy. Yildiz et al. (19) found that the mean baseline serum 25-OH-VitD level was 61 and 49 nmol/L before initiation of and after ASMs therapy, respectively. Such self-control analysis in our study could be finally completed in the 28 of 46 children, and a similar tendency was observed. But this difference was not statistically significant.

Next, we further investigated the effect of different ASMs protocols on serum VitD status. Previous reports (14, 18, 59) have documented that EIASMs increase VitD elimination by stimulating especially CYP3A and CYP24A. However, the effects of NEIASMs, such as VPA, on VitD status remain controversial (15, 17). In our study, there were no significant changes in serum 25-OH-VitD levels at initial baseline and after ASMs treatment. But polytherapy caused significantly lower serum 25-OH-VitD levels than VPA monotherapy. Moreover, only children with epilepsy who received VPA alone or in combination with other ASMs for more than 3 years had serum 25-OH-VitD levels significantly lower than baseline levels (*P* = 0.004; Figures 2, 8). As mentioned earlier, age is an important determinant in serum VitD levels. However, the effect of age on serum VitD in children receiving long-term VPA treatment appeared to play a minor role, and the true effect might be due to the longer treatment duration (Supplementary Figure 3) (54).

Interestingly, among the seven dosing protocols, children treated with VPA monotherapy had the highest median serum 25-OH-VitD levels, and such levels matching deficient or insufficient only accounted about 40% of the VitD test readings. The lowest median value of VitD occurred in the AST-P3 protocol, and the corresponding percentage was as high as 57% yet. In line with our findings, Bergqvist et al. (60) revealed a decreasing by about 17.5 nmol/L in serum 25-OH-VitD levels for each individual add-on ASM in children with epilepsy.

Unexpectedly, there was no any association between serum 25-OH-VitD levels and the plasma concentrations of VPA (Supplementary Figure 4). But Durá-Travé et al. (59) found a clear correlation. The inconsistent results between them partly were caused by the population differences of the enrolled subjects. Thus, VPA appeared to have the weakest impact on serum VitD levels (61), and it could not be concluded that VPA reduces serum 25-OH-VitD levels, which was inconsistent with the results of our previous meta-analysis (16).

The third and most interesting finding is that VitD supplementation not only corrected serum 25-OH-VitD levels in children with epilepsy, but also significantly reduced epileptic activity. A few studies also suggested that seizures could be well controlled by VitD supplementation (22, 62). In this retrospective study, such findings might be further explained from different perspectives of VitD sources.

On the one hand, VitD is mainly produced from sunlight exposure (63–66). Earlier literature involved in animals revealed that VitD played a pivotal role in improving epilepsy seizures (67, 68). It had been proposed that sunlight therapy was one of treatment choices for the epileptics (69–71). Baxendale et al. (71) found that seizures were less likely to occur on bright sunny days. In the current study, it was noted that only in the 3rd quarter, the serum 25-OH-VitD levels in seizure-free cases (median, 58.53 nmol/L) was significantly higher than those in cases who still experienced seizures (median, 49.30 nmol/L, *P* = 0.04). Sunlight is important in the endogenous production and regulation of VitD, and therefore, further studies are needed to investigate the association between sunlight exposure and the serum 25-OH-VitD levels, especially as well as influence on epileptic activity.

On the other hand, as mentioned above, a few studies have reported that direct VitD supplementation could reduce the seizure frequency (22, 62). Christiansen et al. (72) reported VitD supplementation (4,000 IU/day) resulted in a mean seizure frequency reduction by 30% during the treatment. Such study is rare, and the limited studies sometimes have come to inconsistent conclusions. Tombini et al. (20) found no significant reduction in seizure frequency was observed in patients after VitD supplementation, despite significantly elevated serum 25-OH-VitD levels.

In this study, we revealed a clear association between the direct VitD supplementation and the marked increase of serum 25-OH-VitD concentration, and then the significant reduction in seizure frequency (Supplementary Figure 5 and Figure 10). Obviously, there is still lack of authoritative consensus to guide children with epilepsy how to supplement VitD, but some scholars have made positive attempts and provided their own recommendations, like 400 IU/day by Petropoulos et al. (73) and 1,200–3,000 IU/day by Saggese et al. (74). Thus, direct VitD supplementation might present an adjunctive therapy for children with epilepsy receiving long-term ASMs treatments, but more studies investigating the effects of VitD on epileptic activity are warranted, and more critically, evidence-based strategies and guidelines should first be established.

One more question needs to be further discussed. In contrast to a previous study (19), the serum 25-OH-VitD levels were found to be significantly lower in pediatric patients with normal brain MRI findings in our study. Similar finding has been reported by Baek et al. (21). Currently, there is no sufficient evidence to explain the intrinsic association between brain MRI findings and the serum VitD levels. However, it is clear that drug-resistant epilepsy did occur mainly in children with abnormal MRIs. Therefore, to address the underlying mechanisms, more researches are needed in the future.

The strength of this study is dominated by sample sizes, which included 6,338 healthy children and 648 children with epilepsy. This is the largest pediatric epilepsy study to date evaluating VitD status in children on long-term ASMs therapy, meanwhile, this is also a comparative study with serum 25-OH-VitD concentration data from the largest sample size of healthy children. Another strength of the study was that healthy children, children who had just been diagnosed with epilepsy but had not started ASMs therapy, and children who had been on ASMs for different duration, were recruited at the same hospital over the same time period. Obtaining such data could help us reasonably avoid the influence of geographical location, social-economic status, dietary habits, and sunlight intensity when comparing the differences in serum VitD levels among three groups. In addition, as a single-center study, we could determine the 25-OH-VitD levels using the same method and tools through the whole study period, thereby controlling potential bias between them.

However, our study has several limitations due to its retrospective nature. Firstly, the sample size (*n* = 46) of children with epilepsy who met the definition of baseline serum VitD concentration, as well as the sample for self-control analysis (*n* = 35), was too small. This undermines our confidence in reporting the most important findings of this study. Secondly, serum VitD levels in pediatric patients were significantly lower than those in healthy children, which might be associated with disease itself, and ASMs treatment did not continuously worse the VitD levels. However, whether epilepsy caused the reduction in serum VitD levels, or whether the reduction in serum VitD levels caused the seizures, we cannot make a causal judgment. Thirdly, concomitant medication appeared to affect the serum VitD levels, but due to the complexity of drug interactions, we cannot draw certain conclusions about the specific contribution of each individual ASMs therapy. In addition, the limitation due to randomization and eligibility criteria of our retrospective study should also be concerned. Nonetheless, this retrospective study presents a meaningful clinical finding that children with epilepsy have significantly lower serum VitD levels than healthy children, and is helpful for future research into the mechanisms of epilepsy and for improving the childhood epilepsy management.

In conclusion, our retrospective study showed that the proportion of serum VitD deficiency or insufficiency in healthy children was relatively low, but for children with epilepsy, the serum VitD levels significantly decreased. As expected, age, sex, BW, and seasonality became the various determinants of serum VitD levels. Notably, ASM therapy, alone or in combination, did not continuously worse the baseline serum VitD levels in children with epilepsy. The lower serum VitD levels in children with epilepsy than in healthy children might be related to the disease itself, rather than the ASMs treatment. Direct VitD supplementation significantly corrected the serum VitD levels, and was critically associated with a reduction in epileptic activity in those children. However, whether the decrease in serum VitD levels causes epilepsy or whether epilepsy causes the decline in serum VitD levels, we cannot draw a clear conclusion on the causal relationship between them. However, it is a fact that serum VitD levels are significantly reduced in children with epilepsy. Due to the single-center nature, findings obtained in this study might not be generalized to all individuals in China. However, pediatric clinicians are urged to heighten public awareness regarding epilepsy-associated VitD deficiency. To clarify the role of VitD supplementation in children with epilepsy, multi-center and long-term trials with larger sample sizes, as well as basic researches, are necessary in the future.

## Data availability statement

The raw data supporting the conclusions of this article will be made available by the authors, without undue reservation.

## Ethics statement

The studies involving human participants were reviewed and approved by the Ethics Committee of the Children’s Hospital of Nanjing Medical University. Written informed consent from the participants’ legal guardian/next of kin was not required to participate in this study in accordance with the national legislation and the institutional requirements.

## Author contributions

FC, X-PL, and X-NL: concept and design. ND and FC: drafting of the manuscript. FC: critical revision of the manuscript. X-PL and X-NL: administrative, technical, and material support. FC, X-PL, and X-NL: supervision. ND, H-LG, Y-HH, JY, MX, LD, J-CQ, Z-ZJ, FC, X-PL, and X-NL: acquisition, analysis, and interpretation of data. All authors contributed to the article and approved the submitted version.
