# Supplementary material for: Association between serum vitamin D status and the anti-seizure treatment in Chinese children with epilepsy
Source: Front Nutr. 2022 Aug 29;9:968868. doi: 10.3389/fnut.2022.968868 (PMC9464910; doi:10.3389/fnut.2022.968868)
Supplement: Supplementary file 1 [file Data_Sheet_1.DOCX]

**Association between serum vitamin D status and the anti-seizure treatment in Chinese children with epilepsy**

Na Dong^1,2,†,‡^, Hong-Li Guo^1,†^, Ya-Hui Hu^1^, Jiao Yang^3^, Min Xu^4^, Le Ding^4^, Jin-Chun Qiu^1^, Zhen-Zhou Jiang^2^, Feng Chen^1,*^, Xiao-Peng Lu^3,*^, Xiao-Nan Li^5,*^

^1^Pharmaceutical Sciences Research Center, Department of Pharmacy, Children’s Hospital of Nanjing Medical University, Nanjing, China

^2^Institute of Pharmaceutical Science, China Pharmaceutical University, Nanjing 210009, China

^3^Department of Information Science, Children's Hospital of Nanjing Medical University, China

^4^Department of Neurology, Children's Hospital of Nanjing Medical University, Nanjing 210008, China

^5^Department of Children Health Care, Children’s Hospital of Nanjing Medical University, China

†These authors contributed equally to this work.

‡Visiting graduate student from China Pharmaceutical University.

***Corresponding authors:**

Email addresses: [cy.chen508@gmail.com](mailto:cy.chen508@gmail.com) (Feng Chen), [lxp20071113@sina.com](mailto:lxp20071113@sina.com) (Xiao-Peng Lu), and [xiaonan6189@163.com](mailto:xiaonan6189@163.com) (Xiao-Nan Li)

**----Supplemental information----**

**FIGURE LEGENDS**

**FIGURE S1** Correction between age and serum 25-OH-VitD status of healthy subjects and epileptic subjects. (A) Correction between age and serum 25-OH-VitD status of healthy subjects; (B) Correction between age and serum 25-OH-VitD status of children with epilepsy.

**FIGURE S2** Comparison in serum 25-OH-VitD status of male and female subjects with different ages (Infancy, Early Childhood, Middle Childhood and Adolescence). (A) Comparison in serum 25-OH-VitD status of male and female healthy children during the infancy; (B) Comparison in serum 25-OH-VitD status of male and female healthy children during the early childhood; (C) Comparison in serum 25-OH-VitD status of male and female healthy children during the middle childhood; (D) Comparison in serum 25-OH-VitD status of male and female healthy children during the adolescence; (E) Comparison in serum 25-OH-VitD status of male and female epileptic children during the infancy; (F) Comparison in serum 25-OH-VitD status of male and female epileptic children during the early childhood; (H) Comparison in serum 25-OH-VitD status of male and female epileptic children during the middle childhood; (G) Comparison in serum 25-OH-VitD status of male and female epileptic children during the adolescence.

**FIGURE S3** Comparison in serum 25-OH-VitD status of epileptic subjects on VPA treatment with different duration and different ages (Infancy, Early Childhood, Middle Childhood and Adolescence). (A) Proportion of epileptic subjects on different duration of VPA treatment with different ages; (B) Comparison in serum 25-OH-VitD status of epileptic subjects on different duration of VPA treatment during the early childhood; (C) Comparison in serum 25-OH-VitD status of epileptic subjects on different duration of VPA treatment during the middle childhood; (D) Comparison in serum 25-OH-VitD status of epileptic subjects on different duration of VPA treatment during the adolescence.

**FIGURE S4** Correction between serum 25-OH-VitD levels and plasm concentration of VPA.

**FIGURE S5** Comparison in serum 25-OH-VitD status of epileptic children with or without VitD supplementation. (A) Comparison in serum 25-OH-VitD status of all epileptic children with or without VitD supplementation; (B) Comparison in serum 25-OH-VitD status of epileptic children with or without VitD supplementation during the middle childhood.

**Figure S1**

**
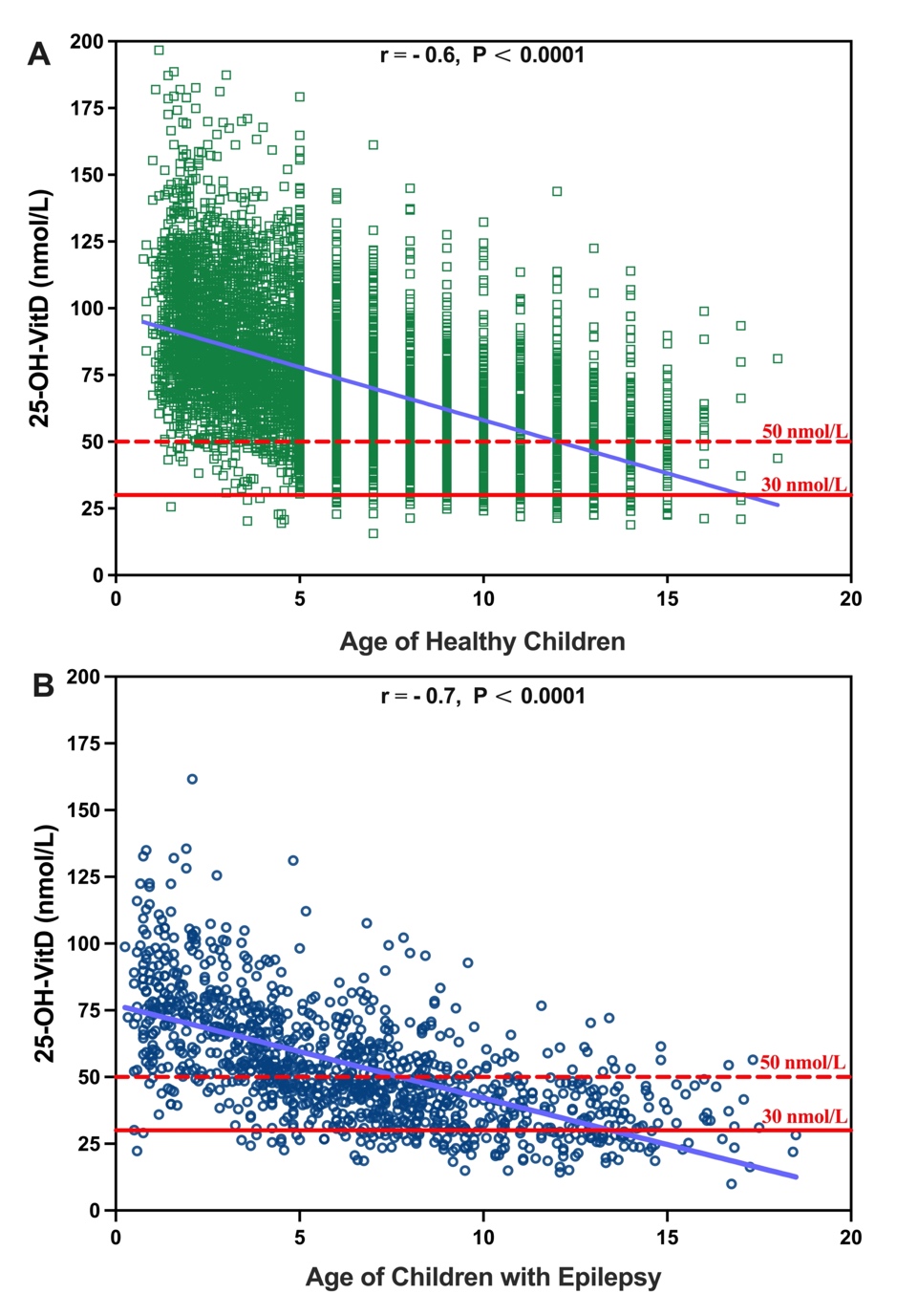
**


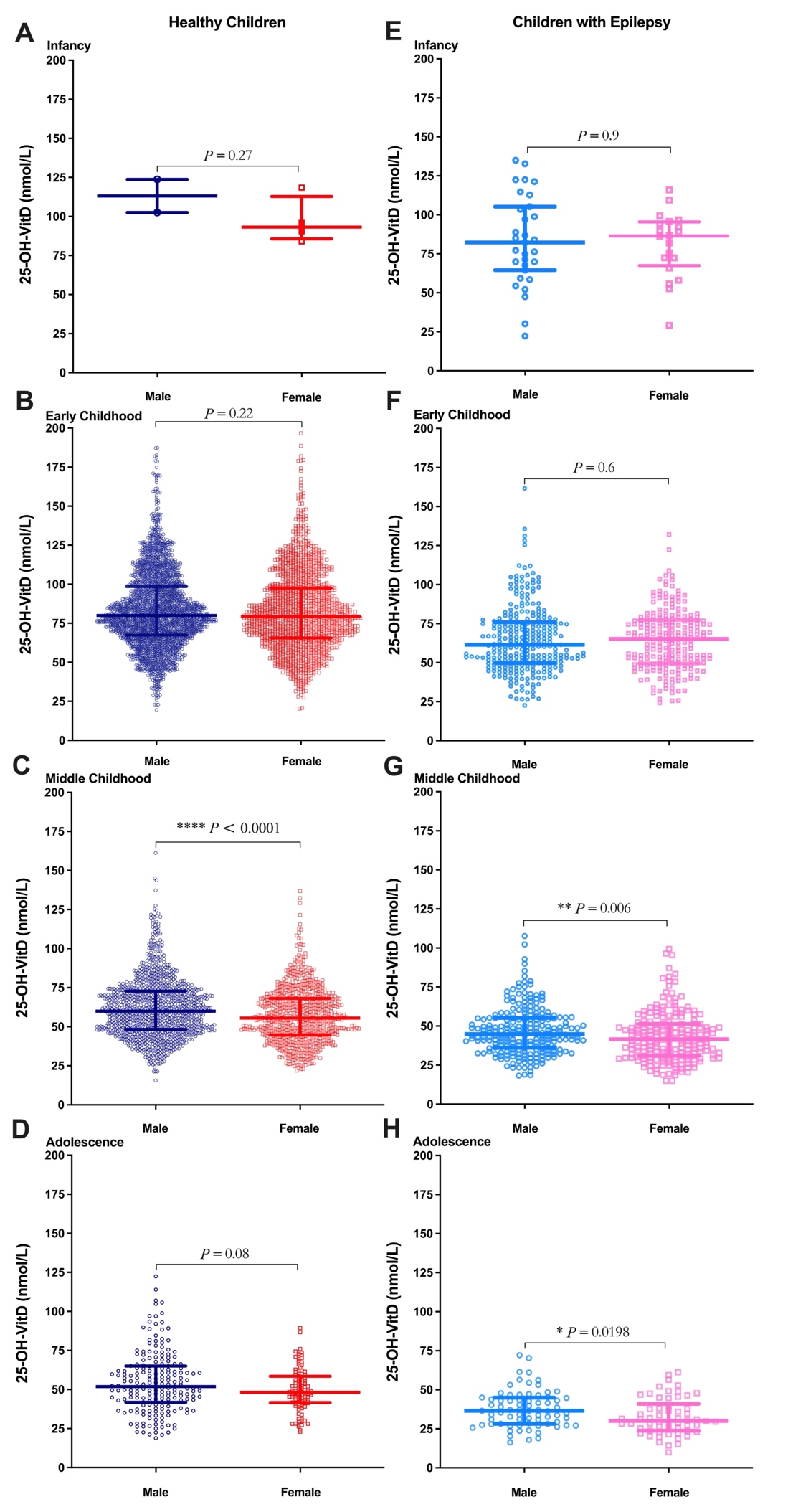
**Figure S2**

**Figure S3**


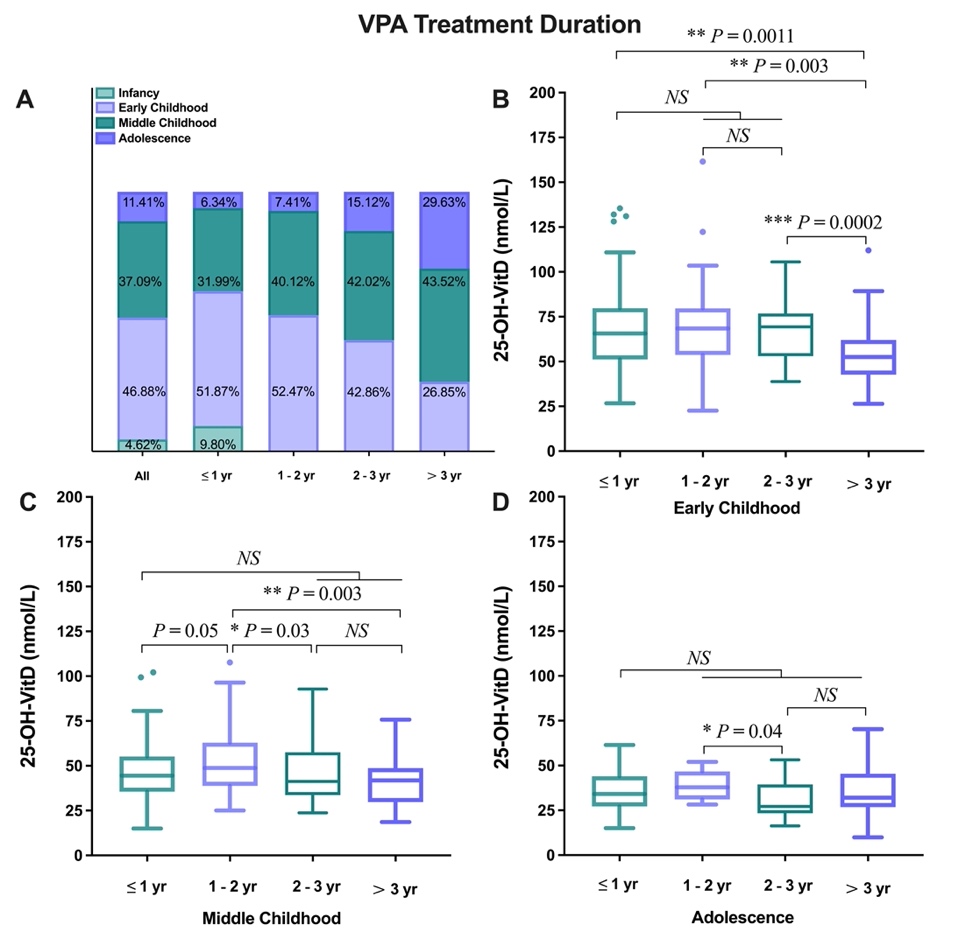


**Figure S4**


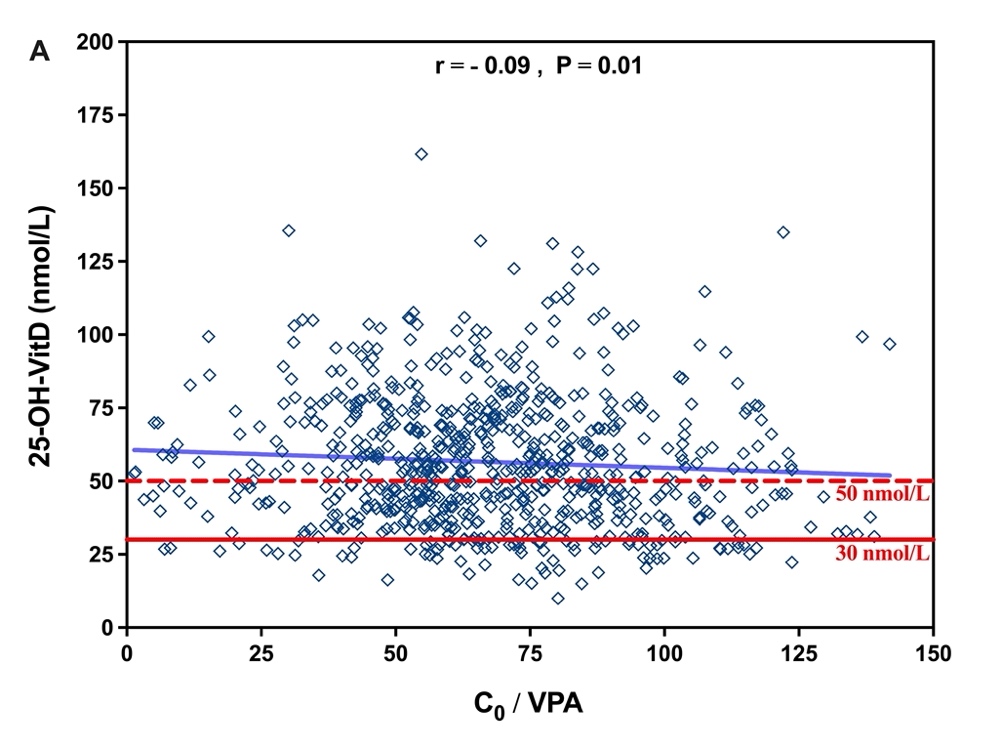


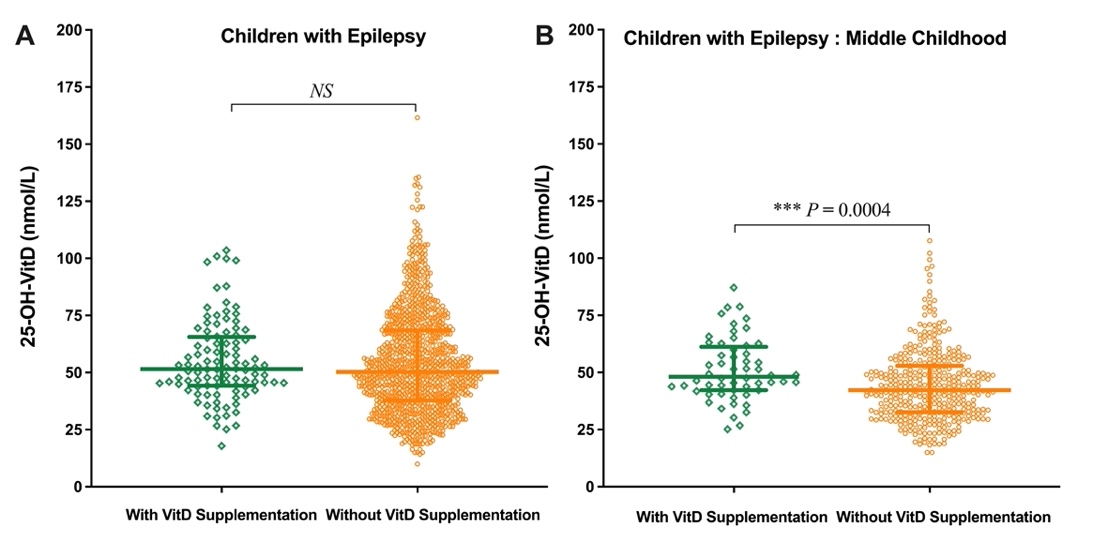
**Figure S5**
